# Influence of head-over-body and body-over-head posture on craniospinal, vascular, and abdominal pressures in an acute ovine in-vivo model

**DOI:** 10.1186/s12987-023-00458-9

**Published:** 2023-08-02

**Authors:** Anthony Podgoršak, Nina Eva Trimmel, Fabian Flürenbrock, Markus Florian Oertel, Margarete Arras, Miriam Weisskopf, Marianne Schmid Daners

**Affiliations:** 1 Department of Mechanical and Process Engineering, ETH Zurich, Zurich, Switzerland; 2 Center for Surgical Research, University Hospital Zurich, University of Zurich, Zurich, Switzerland; 3 Department of Neurosurgery, University Hospital Zurich, University of Zurich, Zurich, Switzerland

**Keywords:** Arterial blood pressure, Cerebrospinal fluid, Hydrocephalus, Intracranial pressure, Sheep model, Transfer function, Tilt testing

## Abstract

**Introduction:**

Optimal shunt-based hydrocephalus treatments are heavily influenced by dynamic pressure behaviors between proximal and distal ends of shunt catheters. Posture-dependent craniospinal, arterial, venous, and abdominal dynamics thereby play an essential role.

**Methods:**

An *in-vivo* ovine trial (n = 6) was conducted to evaluate communication between craniospinal, arterial, venous, and abdominal dynamics. Tilt-testing was performed between –13° and + 13° at 10-min intervals starting and ending at 0° prone position. Mean pressure, pulse pressure, and Pearson correlation (*r*) to the respective angle were calculated. Correlations are defined as strong: |*r*|≥ 0.7, mild: 0.3 <|*r*|< 0.7, and weak: |*r*|≤ 0.3. Transfer functions (TFs) between the arterial and adjacent compartments were derived.

**Results:**

Strong correlations were observed between posture and: mean carotid/femoral arterial (r = − 0.97, r = − 0.87), intracranial, intrathecal (r = − 0.98, r = 0.94), jugular (r = − 0.95), abdominal cranial, dorsal, caudal, and intravesical pressure (r = − 0.83, r = 0.84, r = − 0.73, r = 0.99) while mildly positive correlation exists between tilt and central venous pressure (r = 0.65). Only dorsal abdominal pulse pressure yielded a significant correlation to tilt (r = 0.21). TFs followed general lowpass behaviors with resonant peaks at 4.2 ± 0.4 and 11.5 ± 1.5 Hz followed by a mean roll-off of − 15.9 ± 6.0 dB/decade.

**Conclusions:**

Tilt-tests with multi-compartmental recordings help elucidate craniospinal, arterial, venous, and abdominal dynamics, which is essential to optimize shunt-based therapy. Results motivate hydrostatic influences on mean pressure, with all pressures correlating to posture, with little influence on pulse pressure. TF results quantify the craniospinal, arterial, venous, and abdominal compartments as compliant systems and help pave the road for better quantitative models of the interaction between the craniospinal and adjacent spaces.

## Introduction

Hydrocephalus is characterized by disturbed cerebrospinal fluid (CSF) dynamics [1]. Its treatment has been a topic of debate ever since the first documented use of a valved CSF shunt 72 years ago [2]. Unfortunately, issues that limit the efficacy of shunts, namely subtle pressure fluctuations between the proximal and the distal end of the shunt system, which lead to frequent over- and under-drainage, are to this day not adequately addressed [3]. The ability to design more sophisticated treatment options is limited until gaps in quantitative understanding of CSF dynamics and their communication, in particular, the propagation of pressure from one compartment to another, are filled. Quantifying the interactions between craniospinal, arterial, venous, and intra-abdominal pressures during postural changes can help elucidate the dynamic relationships that exist between these systems, both improving our understanding of the underlying physiology that exists as well as pave the way for improved treatment options to be developed.

The influence of posture on physiological factors, such as intracranial pressure (ICP) [4], functional connectivity [5], cerebral blood flow [6], cerebral venous outflow [7], glymphatic transport of CSF [8], intracranial compliance [9], and cerebral perfusion pressure [10] has been well documented within the literature. In addition, specific investigations into the role of body position on cranial and lumbar CSF pressures in states of normal and impaired craniospinal communication have previously been reported in cats [11]. Furthermore, it is known that body position heavily influences arterial, venous, and respiratory function [12–14].

Previous studies have helped form the foundation of the common physiological understanding of the impact of posture on CSF and its interplay between other anatomical compartments [15–17]. Tilt-tests have been ubiquitously used in clinical and research settings since 1986 when the head-over-body tilt was first described as a non-invasive tool to investigate unexplained syncopes [18]. Exposure of a subject to controlled orthostatic changes in a safe, monitored, laboratory environment, specifically tilt tests are still used today to replicate symptoms and induce autonomic regulatory changes via manipulations to a body position that would otherwise be impossible [19, 20]. Dynamics in the CSF system are governed by the arterial [21, 22], venous [16], and abdominal [23] compartments. Therefore, it is important to acquire a comprehensive quantitative understanding of how these different compartments interact with each other in addition to how they influence intracranial and intrathecal pressures.

Quantifying reactions via mean pressures allows the investigation of bulk pressure reactions and communications within and between compartments [17, 24]. Furthermore, pulse pressure changes provide insights into physiological regulation and system compliances [24]. Transfer functions (TFs) are used to describe the input–output behavior of a system. Additionally, they can be used to model a system and investigate respective dynamics [25, 26]. TFs have previously been used to study dynamic relationships between CSF and cerebral blood flow [27, 28]. However, there exists a research gap in comprehensive studies simultaneously investigating the interplay of intracranial, intrathecal, arterial, venous, and abdominal systems in-vivo.

Thus, in this study, we measured physiological pressure changes of various anatomical compartments simultaneously during tilt testing of anesthetized sheep. The interrelationships are presented and quantified to provide insights into the physiological reactions under gravitationally induced dynamic changes.

## Methods

### Ethical statement

Animal housing and all experimental procedures were approved by the local Committee for Experimental Animal Research (Cantonal Veterinary Office Zurich, Switzerland) under the license number ZH119/2019, conforming to the European Directive 2010/63/EU of the European Parliament and the Council on the Protection of Animals used for Scientific Purposes, as well as to the Guide for the Care and Use of Laboratory Animals [29].

### Anesthesia and animal instrumentation

Six adult female white alpine sheep (age 3.1 ± 1.2 yrs, Body Weight (BW) 75.6 ± 12.1 kg) were included in this study. Anesthesia was induced by intravenous injection of ketamine hydrochloride (Ketasol®-100 ad us. vet.; Dr. E. Graeub AG, Berne, Switzerland; 3 mg/kg BW in combination with midazolam (Dormicum®, Roche Pharma AG, Reinach, Switzerland; 0.2 mg/kg BW and propofol (Propofol®- Lipuro 1%, B. Braun Medical AG; Sempach, Switzerland; 2–5 mg/ kg BW. After intubation, anesthesia was maintained by positive pressure ventilation (12–15 breaths/min, tidal volume 10–15 mL/kg, Fraction of Inspired Oxygen (FiO2) 0.5) of 2–3% isoflurane in oxygen/air mixture and a continuous infusion pump applying propofol (Propofol®- Lipuro 1%, B. Braun Medical AG; Sempach, Switzerland 2–4 mg/kg BW/h). Throughout the procedure, the animals additionally received a continuous intravenous infusion of sufentanil (Sufenta® Forte, Janssen-Cilag AG, Zug, Switzerland; 0.05 mg/kg BW/h). In all sheep, an arterial line (4 Fr) for the measurement of carotid arterial blood pressure (cABP) and a multi-lumen central venous catheter via the jugular vein for the measurement of central venous pressure (CVP) were placed under percutaneous ultrasound guidance. Additionally, in four out of six sheep, a femoral arterial line (10F, Avanti®, Cordis® Corporation, Miami Lakes, Florida, USA) for femoral arterial blood pressure (fABP) measurement and an 18G venous catheter in the proximal jugular vein to measure jugular venous pressure (JVP) were inserted. For measurement of ICP, a 9 Fr catheter (Ref. 55–3000, Neuromedex GmbH, Hamburg, Germany) was placed in the right lateral ventricle, confirmed by CSF egression, and fixed with Ethicon Bonewax (Johnson & Johnson Medical Ltd., Livingston, UK) to avoid CSF leakage. A 4.5 Fr Neuromedex catheter (Ref. 61-1400) was placed in the subdural space to measure intrathecal pressure (ITP) directly at the source via a laminotomy at level L6-7, also sealed with bone wax [30]. Hydrostatic equivalence was maintained between the intraventricular and intrathecal transducers by zeroing them to atmospheric pressure at the level of the lateral ventricles, both arterial and central venous sensors at the right atrial level, and the jugular venous and abdominal sensors at the location of the respective measurement source. Abdominal measurements were acquired directly at the specific abdominal locations via a peritoneal approach with 10 Fr access sheaths (Avanti, Cordis Corporation, Arrow Int. Inc., Reading, PA, USA) at the four different abdominal quadrants: cranial, caudal, dorsal, and ventral (IAPcr, IAPcd, IAPds, IAPve). Abdominal integrity was re-established via the closure of the peritoneum and fascia continuously and the skin via purse-string sutures. Intravesical pressure (IVP) was measured by connecting a transducer directly to the urinary catheter. All transducers were placed at the location of the respective measurement except for the carotid arterial, femoral arterial, and central venous pressures.

**Fig. 1:**
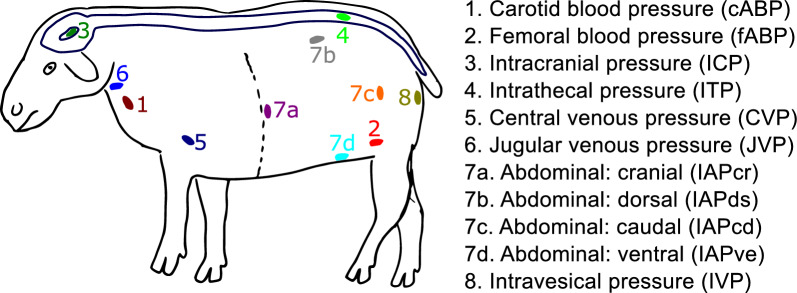
Pressure measurement locations during this study. The color scheme corresponds to the subsequent results

The sheep were then placed in the sternal position throughout the experiment, mimicking the horizontal position in humans at which the ITP and ICP were assumed not to be influenced by hydrostatic variations. Figure 1 shows all sensor locations and pressure signals acquired.

### Experimental protocol

The experimental protocol in this study was designed to reveal how the dynamics between various physiological pressures (Fig. 1) change during standardized changes in body position. Laying on a surgical tilt table in sternal recumbence, body positions of 0°, + 5°, + 10°, + 13°, + 10°, + 5°, 0°, − 5°, − 10°, − 13°, − 10°, − 5°, and 0° were applied; each step was maintained for 10-min. Positive angles denote Head-over-Body (HoB) posture while negative angles indicate Body-over-Head (BoH) posture. The tilt-table operated via a bi-directional electrical actuator with the motor just behind the caudal-most point on the sheep, leading to the fulcrum being around the mid-plane of the sheep to support proper weight distribution throughout the testing.

### Data acquisition, pre-processing, and statistical analyses

All data was acquired using the commercially available software, Ponemah v5.1 (Data Science International, St. Paul, USA) with the ACQ-7700 acquisition unit using the Universal XE and ABCD 4 to amplify the signal. All data was acquired at a sampling frequency of 1 kHz, discriminated to 100 Hz for postprocessing retaining possible high frequency waveform components, and outliers were rejected via a z-score rejection method with a σ_crit_ of 3. Statistical significance of pressure changes was assessed via a paired dependent t-test with tilt angle defined as the independent variable and each pressure signal as dependent. Pressure differences between each step were first averaged across each subject (N = 6) before being entered into the statistical model, yielding results representative of the entire cohort. Significance was determined at a level *of p* < 0.05. In addition, Pearson correlations are reported. Correlations are considered to be strong if |r|≥ 0.7, mild if 0.3 <|r|< 0.7, and weak if |r|≤ 0.3. Regression analysis was performed considering changes in pressure across the entire sheep cohort from the initial mean baseline with all sheep having a complete dataset with no rejections. All analyses were performed using custom scripts in Python 3.7.10 (Open Source, Python Software Foundation, Wilmington, Delaware, USA). The data is available open access at the following link: 10.3929/ethz-b-000623698.

### Mean pressure

**Fig. 2:**
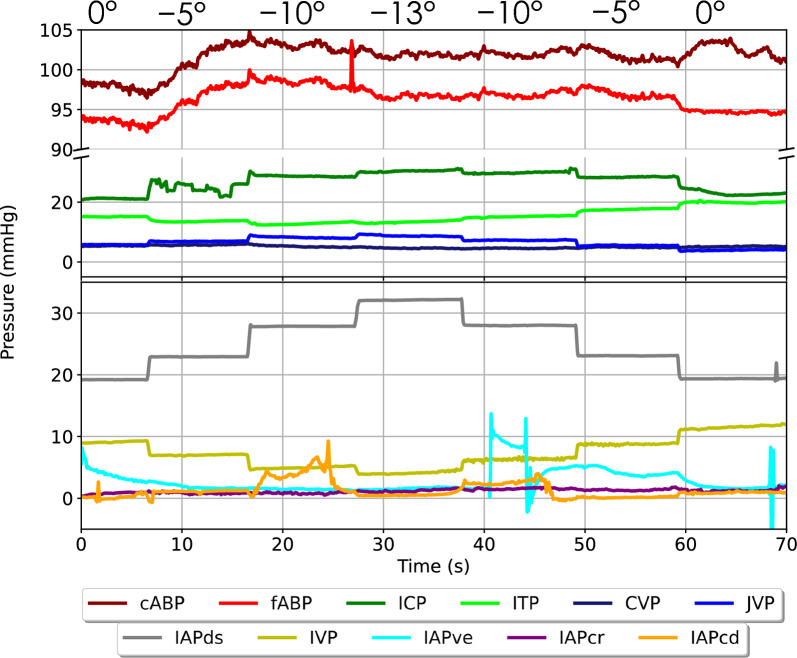
Visualization of mean pressure during body-over-head (BoH) positions in a single sheep; the respective steps are indicated in the range of 0° to − 13° to 0° on top of the figure. Note that head-over-body (HoB) positions are not shown on this figure but were considered in all analyses and that elapsed time is shown on the x-axis. cABP: carotid arterial blood pressure; fABP: femoral arterial blood pressure; ICP: intracranial pressure; ITP: intrathecal pressure; CVP: central venous pressure; JVP: jugular venous pressure; IAPds: dorsal intra-abdominal pressure; IVP: intravesical pressure; IAPve: ventral intra-abdominal pressure; IAPcr: cranial intra-abdominal pressure; IAPcd: caudal intra-abdominal pressure

Mean pressure reactions to different body positions were calculated after the data was pre-processed as follows. To remove effects of the cardiac and respiratory waveforms, the data was lowpass filtered using a 4th order forward/backward Butterworth filter with a cut-off frequency of 0.1 Hz [10]. Mean pressures were defined as 10-min arithmetic means over the entire tilt step once pressures had stabilized (Fig. 2). Note that cABP, fABP, and IAPve change slightly during the initial baseline 0° period: these are normal pressure fluctuations and are considered during the calculation of mean pressure changes.

### Pulse pressure

**Fig. 3:**
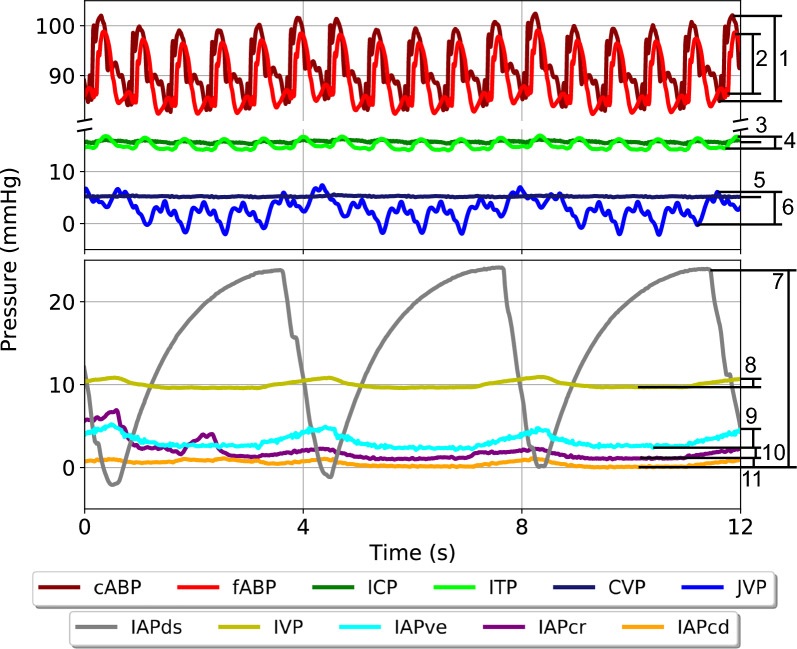
Calculation of pulse pressure. On the right-hand side, the respective measurement (from maximum to minimum) is indicated numerically. cABP_amp_: carotid arterial blood pressure amplitude; fABP_amp_: femoral arterial blood pressure amplitude; ICP_amp_: intracranial pressure amplitude; ITP_amp_: intrathecal pressure amplitude; CVP_amp_: central venous pressure amplitude; JVP_amp_: jugular venous pressure amplitude; IAPds_amp_: dorsal intra-abdominal pressure amplitude; IVP_amp_: intravesical pressure amplitude; IAPve_amp_: ventral intra-abdominal pressure amplitude; IAPcr_amp_: cranial intra-abdominal pressure amplitude; IAPcd_amp_: caudal intra-abdominal pressure amplitude. Additionally, respiratory fluctuations are visible, particularly evident in the abdominal pressures

For all signals, reactions in pulse pressure were calculated as the difference between peak and trough (Fig. 3). Pulse pressures for each waveform were calculated and then averaged over the entire length of the tilting step.

### Transfer functions

Transfer functions (TFs) were calculated by dividing the output and the input signal in the frequency domain. As the heart governs all vascular pressures in the body, cABP was defined as the input and all other pressure signals, such as ICP, ITP, or CVP as the individual outputs. The conversion between time-series to frequency domain was done via discrete fast Fourier transformation (FFT) over the 0°, + 13°, and − 13° steps with a rectangular 10-min window without overlap. Each TF was then smoothed using Gustaffson’s method to avoid transients at the edges of the signal while retaining trends [31]. These results were then averaged across all six sheep to represent our cohort's final TFs. A 95% confidence interval was plotted in addition to the averaged TF. The full 1 kHz data resolution was used in the derivation of each TF to ensure the highest spectral resolution possible.

## Results

### Mean reactions

**Fig. 4:**
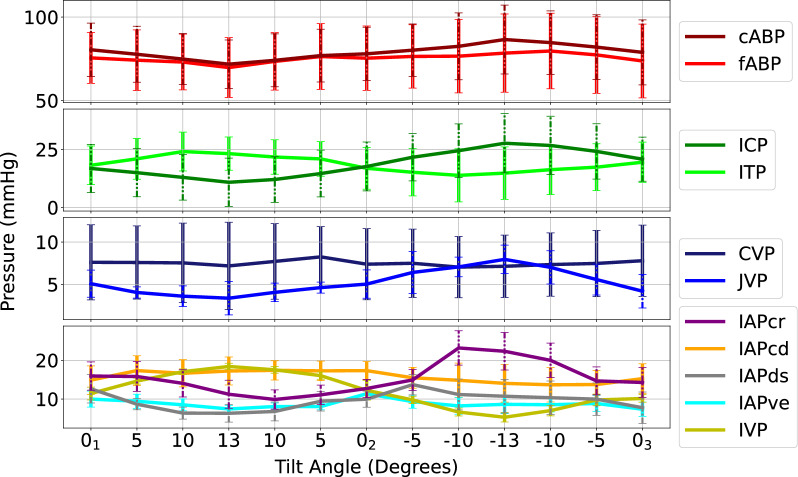
Visualization of mean changes across the entire range of tilt angles and all pressures considered (N = 6). Error bars indicate one standard deviation at each tilt angle. cABP: carotid arterial blood pressure; fABP: femoral arterial blood pressure; ICP: intracranial pressure; ITP: intrathecal pressure; CVP: central venous pressure; JVP: jugular venous pressure; IAPcr: cranial intra-abdominal pressure; IAPcd: caudal intra-abdominal pressure; IAPds: dorsal intra-abdominal pressure; IAPve: ventral intra-abdominal pressure; IVP: intravesical pressure

cABP and fABP show negative correlations with tilt angles, i.e., as tilt angle increases, mean pressure decreases (Table 1). ICP and ITP (horizontal anatomic distance from ITP to ICP sensor locations of 70.0 ± 3.2 cm, respective vertical distance of 0.4 ± 0.8 cm) reacted opposingly to each other, highlighting their hydraulic connection via the spinal canal. Minimal reaction and mildly positive correlation in CVP were observed while JVP revealed a similar strongly negative correlation with tilt angle as cABP and fABP. There were less obvious patterns with IAPs, however a mild direct relationship with tilt angle can be observed in IAPcd (horizontal anatomic distance from IAPcd to ICP sensor locations of 60.5 ± 4.3 cm, respective vertical distance of 9.5 ± 5.5 cm) and IVP (horizontal anatomic distance from IVP to ICP sensor locations of 76.4 ± 5.2 cm, respective vertical distance of 16.8 ± 3.6 cm) and a mild indirect relationship in IAPcr (horizontal anatomic distance from IAPcr to ICP sensor locations of 56.7 ± 5.6 cm, respective vertical distance of 0.9 ± 2.6 cm), IAPds (horizontal anatomic distance from IAPds to ICP sensor locations of 61.3 ± 4.7 cm, respective vertical distance of − 3.7 ± 2.7 cm), and IAPve (horizontal anatomic distance from IAPve to ICP sensor locations of 62.7 ± 4.8 cm, respective vertical distance of 7.3 ± 3.5 cm) (Fig. 4). Equal and opposite correlations were observed in IAPcr and IAPcd, while IVP yielded a strong correlation to tilt (*r* = 0.99). A positive vertical distance denotes when the respective sensor transducer is below the ICP transducer, and negative when above the ICP transducer.

**Table 1:** Mean pressure changes over the six sheep from the first baseline prone position (0_1_°) listed first as averaged values then as the respective relative values from 0_1_

Angle (˚)		cABP (mmHg)	fABP (mmHg)	ICP (mmHg)	ITP (mmHg)	CVP (mmHg)	JVP (mmHg)	IAPcr (mmHg)	IAPcd (mmHg)	IAPds (mmHg)	IAPve (mmHg)	IVP (mmHg)
0_1_	Mean Pressure	80.4 ± 16.2	75.5 ± 17.7	16.8 ± 9.7	18.2 ± 8.2	7.6 ± 4.0	5.1 ± 1.6	15.9 ± 3.2	14.9 ± 1.7	12.6 ± 0.8	10.0 ± 0.4	11.4 ± 3.2
+ 13	Δ	− 8.6 ± 5.8	− 4.6 ± 5.8	− 5.9 ± 3.3	+ 5.0 ± 3.2	+ 0.4 ± 4.3	− 1.7 ± 3.1	− 4.7 ± 3.1	+ 2.4 ± 7.4	− 6.3 ± 18.9	− 2.6 ± 6.2	+ 7.0 ± 1.9
0_2_	Δ	− 2.4 ± 6.2	− 0.3 ± 2.5	+ 0.9 ± 3.7	− 1.3 ± 3.4	− 0.1 ± 1.9	+ 0.0 ± 0.6	− 3.2 ± 6.8	+ 2.4 ± 6.8	− 3.7 ± 10.5	+ 1.4 ± 4.5	+ 0.8 ± 2.1
− 13	Δ	+ 6.1 ± 8.7	+ 1.5 ± 7.3	+ 10.8 ± 5.6	− 3.4 ± 4.9	− 0.1 ± 1.6	+ 2.9 ± 1.9	+ 6.4 ± 20.6	− 0.8 ± 8.4	− 1.9 ± 22.3	− 1.3 ± 2.2	− 6.1 ± 2.9
0_3_	Δ	− 1.5 ± 6.0	− 1.0 ± 5.4	+ 4.0 ± 5.2	+ 1.3 ± 3.9	− 0.1 ± 2.6	− 0.9 ± 1.9	− 1.7 ± 12.5	+ 0.4 ± 4.1	− 4.9 ± 22.8	− 2.6 ± 2.8	− 1.3 ± 2.3
*r*		− 0.97	− 0.87	− 0.98	0.94	0.65	− 0.95	− 0.83	0.84	− 0.73	− 0.19	0.99
*P-*value		*.036*	*.014*	*.030*	*.036*	*.045*	*.047*	*.044*	*0.032*	*.014*	*.030*	*.031*

Angle (˚)		cABP_amp_ (mmHg)	fABP_amp_ (mmHg)	ICP_amp_ (mmHg)	ITP_amp_ (mmHg)	CVP_amp_ (mmHg)	JVP_amp_ (mmHg)	IAPcr_amp_ (mmHg)	IAPcd_amp_ (mmHg)	IAPds_amp_ (mmHg)	IAPve_amp_ (mmHg)	IVP_amp_ (mmHg)
0_1_	Mean Amplitude	18.8 ± 6.6	26.0 ± 9.6	2.6 ± 1.2	3.0 ± 1.1	3.7 ± 2.0	1.2 ± 0.8	7.7 ± 7.6	4.6 ± 3.9	11. ± 10.2	6.2 ± 7.2	1.7 ± 0.8
+ 13	Δ	− 0.7 ± 5.2	− 3.2 ± 6.1	− 0.1 ± 0.6	− 0.4 ± 1.1	+ 0.2 ± 1.7	− 0.5 ± .05	− 5.1 ± 8.0	− 1.8 ± 7.1	− 9.0 ± 10.9	− 3.6 ± 7.8	+ 0.0 ± 0.2
0_2_	Δ	+ 3.0 ± 2.5	− 1.9 ± 4.7	+ 0.4 ± 0.8	− 0.5 ± 0.8	+ 1.1 ± 1.6	+ 0.2 ± 0.3	+ 0.7 ± 5.1	− 3.2 ± 6.9	− 2.8 ± 18.7	− 1.7 ± 4.9	+ 0.0 ± 0.4
− 13	Δ	+ 0.3 ± 8.2	− 1.5 ± 5.1	+ 0.9 ± 0.9	− 0.3 ± 0.7	+ 0.1 ± 1.2	+ 0.9 ± 1.3	− 6.2 ± 9.8	− 2.4 ± 7.0	− 7.9 ± 9.6	− 2.1 ± 3.8	− 0.3 ± 0.4
0_3_	Δ	+ 2.1 ± 2.3	− 3.9 ± 4.8	+ 0.7 ± 1.1	+ 0.0 ± 0.8	+ 0.3 ± 1.0	+ 0.5 ± 0.8	− 4.4 ± 6.7	− 2.7 ± 6.5	− 10.5 ± 10.8	− 3.4 ± 7.1	− 0.6 ± 0.7
*r*		− 0.49	0.31	− 0.91	0.16	− 0.07	− 0.93	0.15	0.37	0.21	− 0.01	0.66
*P-*value		*.292*	*.232*	*.434*	*.465*	*.488*	*.459*	*.217*	*.168*	*.025*	*.183*	*.471*

(+ 13°, 0_2_°, − 13°, 0_3_°) for cABP: carotid arterial blood pressure; fABP: femoral arterial blood pressure; ICP: intracranial pressure; ITP: intrathecal pressure; CVP: central venous pressure; JVP: jugular venous pressure; for IAPcr: cranial intra-abdominal pressure; IAPcd: caudal intra-abdominal pressure; IAPds: dorsal intra-abdominal pressure; IAPve: ventral intra-abdominal pressure; and IVP: intravesical pressure as well as mean pulse pressures for cABP_amp_: carotid arterial blood pressure amplitude; fABP_amp_: femoral arterial blood pressure amplitude; ICP_amp_: intracranial pressure amplitude; ITP_amp_: intrathecal pressure amplitude; CVP_amp_: central venous pressure amplitude; JVP_amp_: jugular venous pressure amplitude; IAPcr_amp_: cranial intra-abdominal pressure amplitude; IAPcd_amp_: caudal intra-abdominal pressure amplitude; IAPds_amp_: dorsal intra-abdominal pressure amplitude; IAPve_amp_: ventral intra-abdominal pressure; and IVP_amp_: intravesical pressure. All values are in mmHg (mean ± SD) with Pearson correlation (*r*) and p-values reported. Δ denotes a change in pressure from the angle relative to the first baseline (0_1_°)

Table 1 shows the relative changes in mean pressures at 0° baseline prone position (0_1_°) and the maximum angle (+ 13°) and minimum angle (− 13°). Additionally, relative changes between baseline (0_1_°) second prone 0_2_° and third prone position 0_3_° are shown. Similar behaviors in cABP and fABP were observed, where both decrease at + 13°, followed by an increase at − 13°. cABP and fABP remained decreased upon the return to 0° (0_2_° and 0_3_°) compared to the first 0° baseline 0_1_°. ICP and ITP as well as IAPcr and IAPcd showed opposing reactions to each other i.e., as IAPcr increased, IAPcd decreased, highlighting their 180° measurement directions. IAPds and IAPve did not show opposing behavior as observed in the cranial and caudal measurements. IVP showed similar behavior to IAPcd. IAPcr, IAPcd, and IVP all returned to values within one SD of the original baseline while IAPds and IAPve were considerably below their original baseline pressure value. A large standard deviation was observed across the entire range of tilt angles in IAPds.

All changes in mean proved to be statistically significant, with cABP, ICP, and JVP, IAPcr, and IAPds all being strongly negatively correlated to tilt. CVP proved weakly positively correlated, and ITP being strongly positively correlated with tilting angle. IAPcd and IVP proved to be strongly positively correlated with tilting angle. In all pressures, disproportionate reactions to tilt angle were observed, especially in ICP, with + 13° yielding a pressure change of − 5.9 mmHg while − 13° yielded a pressure change of 10.8 mmHg compared to the initial baseline.

### Pulse pressure reactions

**Fig. 5:**
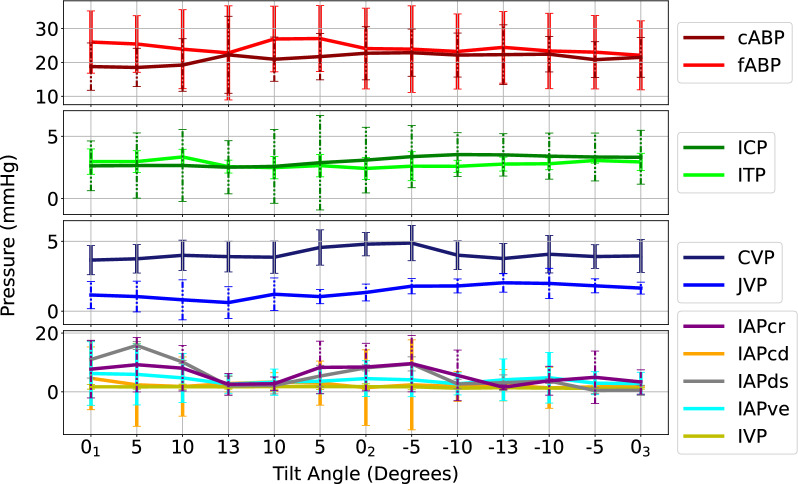
Visualization of pulse pressure changes across the entire range of tilt angles and all pressures considered (N = 6). Error bars indicate one standard deviation at each tilt angle. cABP, carotid arterial blood pressure; fABP, femoral arterial blood pressure; ICP, intracranial pressure; ITP: intrathecal pressure; CVP: central venous pressure; JVP jugular venous pressure; IAPcr: cranial intra-abdominal pressure; IAPcd: caudal intra-abdominal pressure; IAPds: dorsal intra-abdominal pressure; IAPve: ventral intra-abdominal pressure; IVP: intravesical pressure

Less noticeable patterns and weaker correlations were observed in pulse pressure amplitudes (Fig. 5). A slight increase in cABP_amp_ from 0° to + 13° corresponded with a decrease in fABP_amp_ over the same time window. There were slight changes in ICP_amp_ and ITP_amp_ over the range of tilt angles, however they lacked significance. CVP_amp_ and JVP_amp_ remained relatively constant across the entire range. From 0° to + 5°, there was an increase in IAPcr_amp_ and IAPds_amp_ amplitude with a decrease of IAPcd_amp_ and IAPve_amp_. However, for the remainder of the tilt test, all IAPs followed a similar pattern between each other while IVP remained relatively constant across the entire range. Only changes seen in IAPds_amp_ proved to be statistically significant.

### Transfer functions

**Fig. 6:**
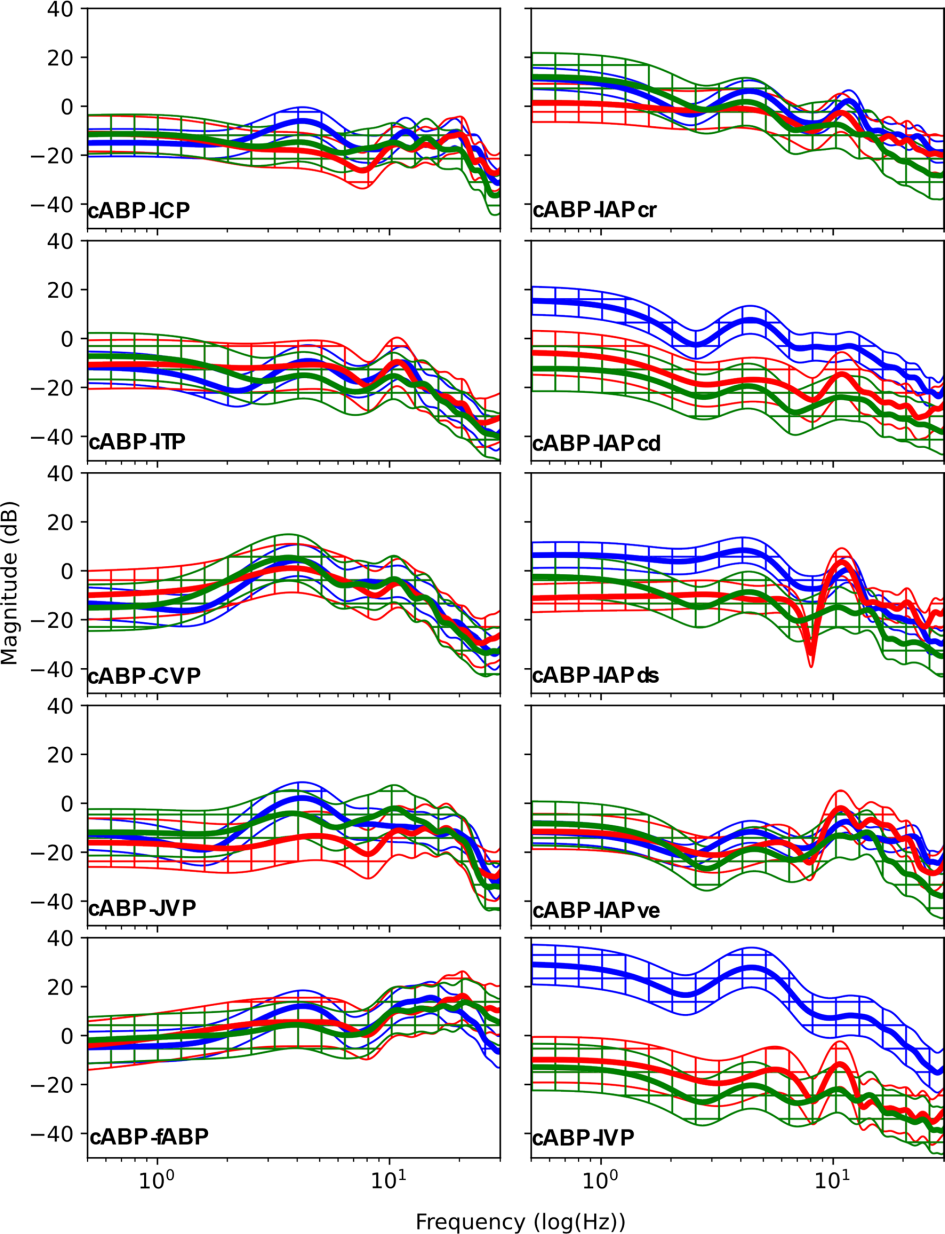
Transfer functions (TF) of all measured pressures averaged over all sheep (N = 6). Blue, initial prone position 0_1_°; red, + 13°; and green, − 13° tilt angles. A 95% confidence interval is plotted following the same color scheme. All TFs are computed with the carotid arterial blood pressure (cABP) as the input to each individual output: ICP: intracranial pressure; ITP: intrathecal pressure; CVP: central venous pressure; JVP: jugular venous pressure; fABP: femoral arterial blood pressure, IAPcr: cranial intra-abdominal pressure; IAPcd: caudal intra-abdominal pressure; IAPds: dorsal intra-abdominal pressure; IAPve: ventral intra-abdominal pressure; and IVP: intravesical pressure

Dynamic transmission patterns were observed across all TFs with considerably different magnitudes across 0°, + 13°, and − 13° (Fig. 6). At 0° in the arterio-craniospinal, arterio-venous, and arterio-abdominal TFs, there exist an initial soft decay; however, this observation is not as strong in the + 13° and − 13° TFs. Following this initial behavior there exist dynamic resonant peaks at 4.2 ± 0.4 and 11.5 ± 1.5 Hz before a final decay with roll-offs of − 14.9 ± 5.3, − 17.0 ± 4.3, − 17.0 ± 3.2, − 15.1 ± 6.3, − 14.3 ± 7.2, − 16.2 ± 6.5, − 14.9 ± 5.5, and − 17.7 ± 2.9 dB/decade for cABP-ICP, cABP-JVP, cABP-IAPcr, cABP-IAPcd, cABP-IAPds, cABP-IAPve, and cABP-IVP, respectively. These local minima and maxima are more prevalent in the arterio-venous and arterio-CSF TFs when compared to the arterio-abdominal TFs.

## Discussion

### Pressure responses

Across the entirety of the tilt tests, ICP and ITP revealed opposite reactions to the orthostatic changes. ICP was in a strong indirect relationship with the tilt angle causing a decrease in mean ICP as the angle became more HoB. This hydrostatic change may have caused pressure to drop via a gravitationally induced relaxation of the cerebrovascular bed and an increase in compliance. Conversely, as the sheep becomes more BoH, CSF and venous blood pool in the cranium, causing an increase in their mean pressure. Similar volumetrically-induced changes in pressure dynamics have previously been reported [21]. Interestingly, ICP and ITP did not return to their pre-tilt baseline, ending slightly higher. This may indicate an autonomic compensation via the orthostatic stressing of the CSF [4] and glymphatic system leading to a temporary reduction in CSF absorption, causing increased pressure post-tilt test. The order of the tilt test (i.e., starting with BoH rather than HoB) may further impact the final return to baseline. The influences of these different positions are split between gravitational influences and the resultant volumetric pressure changes. However, intracranial and lumbar intrathecal spaces have similar relaxation times when a volumetric pressure change is introduced [24]. Furthermore, recent work has shown that there exists similar venous dynamics in sheep and humans in response to tilt [32, 33], therefore there may be a level of venous collapse that effectively reduces CSF outflow during upright postures, maintaining ICP and reducing the influence of HoB posture, as also supported by the influence as dictated by the ICP-ITP hydrostatic column (15.7 cm at ± 13°, calculated using trigonometry, leading to 11.6 mmHg) being suggesting a larger pressure change than what was observed. Therefore, these findings suggests that gravitational influences may cause uneven dynamic changes in HoB and BoH postures.

Both cABP and fABP responded similarly to the orthostatic influence on ICP, but most likely induced via different mechanisms. Vasovagal and baroreflex responses have been well described in humans to maintain stable hemodynamics following a change in body position [34, 35, 35, 36, 36, 37]. Furthermore, baroreceptor activity and venous dynamics when compared between newborn sheep and humans have been shown to be functionally similar [33, 38]. As gravity’s directional influence on the cardiovascular system changes, baroreceptors create a negative feedback loop to maintain homeostasis and regulate the vasovagal syncope caused by tilting. Sato et al. [39] reported in humans that baroreflex is heavily attenuated under propofol-based anesthesia, which may motivate the apparent posture-induced changes to cABP and fABP in our sheep. There was minimal change in CVP across the range of tilts. As CVP dynamics are usually dictated by cardiopulmonary blood volume, perhaps the ± 13˚ extremities were insufficient to provide this volume change potentially due to its location near its respective hydrostatic indifference point. JVP, however, displayed a clear indirect relationship with tilt angle similar to the arterial pressures and ICP. In humans, a split jugular venous system exists comprised of the internal and external jugular veins connected to the brachiocephalic vein. The internal jugular vein is thereby described to collapse during upright posture to regulate ICP via an increase in venous outflow resistance [16, 40]. This, in turn, causes the vertebral venous plexus to act as the primary venous outflow tract while upright [41]. However, in sheep there lacks an internal jugular vein, potentially leading to the vertebral plexus playing a more important role in physiological compensation to orthostatic variations than in humans, causing the more muted response in JVP due to relatively more venous drainage occurring in the vertebral veins.

The simultaneous measurement of IAP while undergoing tilting is less described in the literature, with only a few studies reporting changes in compartmental IAP following trauma and liver transplantation [23, 42, 43]. Our study motivates the idea that the intra-abdominal space is heterogeneous and should not be considered a unified entity. IVP is commonly used as a non-invasive indirect measurement of IAP in patients. Reasonable agreement, however, was only found between IAPcd and IVP in our sheep, thereby questioning the validity of IVP particularly in light of VP shunt placement and the flexibility and mobility of the distal shunt catheter, which is commonly implanted via an incision in the upper abdomen of patients, more cranially than the caudal sensor placement in our sheep cohort. A significant variation in IAP measured in the upper and lower abdominal compartment following liver transplantation in humans aggravated by postural changes has previously been described by Cresswell et al. [43]. We found IAPcr and IAPds to have negative correlations with tilt angle while IAPcd, IAPve, and IVP were observed to be positively correlated. Sheep, like other ruminants, have a highly asymmetric abdominal compartment, with the rumen occupying nearly all of the left side [44]. It might therefore be assumed that the regional IAP effect may not be as prominent in humans. However, this makes sheep unique in the development and testing of novel shunts, as potentially varying pressure gradients between proximal and distal ends of the shunt can be studied to improve shunt function. Furthermore, these ovine results motivate a deeper investigation into the concept of a unidimensional IAP measurement as it may fail to describe true IAP dynamics adequately.

These results, while showing distinct dynamics between the proximal (craniospinal) and distal (arterial, venous, or abdominal) ends of any shunt catheter, emphasize the correspondent paired behavior in the craniospinal, arterial, and venous compartments while also visualizing the heterogeneity of the abdominal space.

### Pulse pressure responses

Whenever the CSF space and surrounding area endure a volumetric fluid increase, a reduction in compliance occurs due to the stretching of the cavity walls as a reaction to the increase in pressure, e.g. brain compliance is inversely proportional to ICP [45]. As CSF is dynamically pulsatile, the fluid’s pulse pressure will also change as the cavity becomes less elastic [46]. In our study, gravitational forces acting upon the sheep lead to flooding of the venous bed and cerebral intraventricular space (in the case of BoH) and potentially a rush of CSF pooling in the lumbar regions (in the case of HoB). During BoH, cranial pooling of CSF causes the surrounding ventricular tissue to experience an acute reduction in compliance, leading to an increase in ICP_amp_ and a corresponding decrease in ITP_amp_. Oppositely, during the HoB position, CSF pools in the lumbar regions, causing an increase in ITP_amp_ and a decrease in ICP_amp_, possibly following a reduction of lumbar compliance. As CVP remained relatively constant during tilting the pulse pressure also remained relatively stable. In JVP, a large dynamic change in mean pressure was observed, with no corresponding increase in pulse pressure. As the jugular vein is a high-compliance vessel, it is possible that the extent of tilting may not have induced enough of a volumetric change to effectively cause a reduction in compliance, leading to increased pulse pressures.

No obvious patterns were observed in the arterial pulse pressures across tilt angles, which may be a manifestation of our − 13° to + 13° tilt steps. Furthermore, the arterial tree is a muscular system, which can counteract gravitational pooling, in contrast to the CSF and venous systems. This effect suggests a certain amount of independence between ABP_amp_ and posture. No distinct pulse pressure patterns were observed in the abdominal pressures, some behavior even disagreed with the changes in mean pressure e.g., large changes in pulse pressure that are yet to have a physiological explanation. This may be attributed to the heterogeneity of the sheep's abdominal cavity. The ovine rumen is filled very specifically with fluid and solid matter on the bottom, followed by a layer of solid matter in the middle and gas on the very top. It is possible that the tilting of the sheep changed the layering of the ruminal content and caused the position of the abdominal organs to shift—both of which may have led to the seemingly spontaneous changes in pulse pressure. Overall, these results illustrate the fact that there exists minimal marked impact from tilting on pulse pressures in the craniospinal, arterial, venous, and abdominal spaces.

### Transfer function analysis

The classical view of large-artery compliance is that it supports the dampening of strong cardiac ventricular ejections and assists in the reduction of the pulsatile nature of the flow into a more constant downstream flow at the site of the arterioles, thus supporting organ perfusion and ensuring consistent and manageable pulsatility to the downstream vessels [47]. In a similar physiological phenomenon, intracranial compliance has been described as essential to the compensatory mechanisms to maintain ICP stability and intracranial homeostasis [48].

In a mathematical sense, compliant mechanisms operate by attenuating higher frequencies—i.e., they act as lowpass filters. The general lowpass behavior of our derived TFs motivates the physiological doctrine of arteries, veins, the craniospinal space, and the abdomen behaving as compliant compartments. Furthermore, multiple resonant peaks (local maxima) exist among each TF, such as the cABP-ICP, cABP-ITP, or cABP-fABP TF. The existence of complex yet correspondent resonant peaks in the arterio-craniospinal and arterio-venous TFs suggests that these system responses exhibit common features due to resonance, agreeing with the work of Wagshul et al. [49] yet disagreeing with the work of Tenti et al. [50], who argued that resonance plays no role in the synchronicity of arterial and CSF pulsations.

Interestingly, there exists behavior indicative of a notch filter at 8.0 Hz in cABP-IAPds and to a lesser extent in cABP-IAPve and cABP-IVP. Notch behavior has previously been reported [49], however it was only reported in cABP-ICP TFs. The TFs remained quantitatively similar across the three tilt angles (0°, + 13°, − 13°) with few exceptions. cABP-IAPcd and cABP-IAPds TF magnitudes at 0° remained asymmetrically larger than their + 13° and − 13° counterparts until around 9 Hz. + 13° and − 13° were the two tilt extrema considered in this study and IAPcd and IAPds were facing the tail and back of the sheep, respectively. As introduced prior, the ovine rumen is comprised of transient layers. If an asymmetric shift in the rumen content occurred, this might have manifested itself in a similar asymmetric manner to the different abdominal pressure measurements. This could, in turn, have caused a corresponding asymmetric shift in the individual TFs between 0°, + 13°, and − 13°, which may also explain the cABP-IVP TF remaining asymmetrically larger than the + 13° and − 13° ones. Each of our TFs final decays are within the range of 20 dB/decade, which may lead to the assumption that first-order lowpass behavior dominates. However, bands of local maxima and minima (i.e., resonance) before the final decay suggest that some intercompartmental interactions are subject to higher order dynamics, possibly due to the existence of multiple physiological pathways from the carotid artery to the craniospinal, venous, and abdominal spaces. Nevertheless, these results show that we have similar transmission patterns across the investigated compartmental pairs and that there does exist attenuation of higher frequencies. This supports the concept that the arterial, craniospinal, venous, and abdominal systems serve as compliant mechanisms.

The derivation of TFs between the carotid arterial and adjacent compartments has revealed complex compliant systems which are dictated by multiple overlain higher-order dynamics.

### Clinical and physiological implications

The optimal strategy of shunt-based treatments for hydrocephalus requires a detailed understanding of the dynamic pressure behaviors that exist between proximal and distal ends of the shunt catheters (i.e., intraventricular and peritoneal abdominal spaces for ventriculoperitoneal shunts, intraventricular and venous spaces for ventriculo-venous shunts, and intraventricular and atrial cardiac spaces for ventriculo-atrial shunts) [51]. This study provides a foundation to build this understanding via analyzing the dynamic reactions to tilting. Observing the impact of tilt angle on physiological pulse pressures is an important consideration when designing the control strategy behind any active shunts—specifically to avoid over- or under-drainage at the systolic peak or diastolic trough. This study shows that moderate HoB and BoH tilt have minimal impact on pulse pressures, meaning that, at least from − 13° to + 13° of postural change, there need not exist unique sophistications to adjust for changes in pulse pressure. However, an expansion of this investigation to the entire range of normal body postures (0° to 90°) is required. There was no indication of reflex tachycardia during HoB positions, which does not necessarily suggest that the reflex pathway is not present, rather perhaps that our moderate tilt angles were insufficient to trigger them. Moreover, the effect of posture on glymphatic drainage has also been a topic of interest in the field, with one study conducted in rats showing that the lateral sleeping position led to the most optimal glymphatic drainage when compared to the supine and right lateral decubitus positions [52]. It could also be the case that, depending on the brain’s position relative to the fulcrum of the tilt, glymphatic drainage may vary. Furthermore, the compliant nature of most body parts has been a topic of considerable discussion ever since Spencer and Denison [53] first quantified arterial compliance in 1963. The derived TFs have quantified the intercompartmental compliant behavior, revealing which specific frequencies are attenuated to what extent. Moreover, having the TFs between arterial, venous, craniospinal, and abdominal compartments supports the investigation into specific behaviors at all possible spectral inputs, paving the way for a more quantitative understanding of how compliance plays a role in physiology.

### Physiological considerations

This investigation was performed only in female sheep; however, no sex-based pressure differences are anticipated. A balanced, multimodal anesthesia was used, with the intention to reduce the dose of each anesthetic agent and thus their side effects. General anesthesia is known to affect autoregulatory mechanisms to a certain extent, which is common limitation in physiological studies performed under general anesthesia. However, due to the nature of manipulations performed in this experiment, general anesthesia was necessary and was considered in the interpretation of all findings. Moreover, our acute model under anesthesia ensures no nodding of the head during movements, effectively avoiding head-movement-induced hydrostatic influences.

### Limitations

The use of mechanical positive pressure ventilation could not only have had an impact on venous and thoracic pressure but also on the flow of CSF, which may have influenced our results. Furthermore, as sheep are quadrupeds, elements specific to their physiological fluid dynamics limit the transferability to humans. Specifically, quadrupeds are physiologically adapted to changing CSF axis closer to the neck when compared to humans where the CSF axis is firstly vertical most of the time, and secondly much more prone to larger changes. This may also lead to limitations in translating CSF-specific relationships to humans. The ovine abdominal space is anatomically different from that of a human, with multiple different layers comprised of different contents and ruminal filling stages. The effects suspected to have been caused by regional abdominal heterogeneities observed in this study may not be as distinct in humans, whose abdominal anatomy is different and not as complex as the ovine system. The tilt-table used in this study was limited to ± 13˚ in a prone position, which might limit conclusions and direct comparisons to human body positions, such as standing, sitting, or supine.

## Conclusions

Our investigations have provided comprehensive insights into the dynamic physiological relationships between the pressure conditions at the proximal and distal positions of any shunt catheter during changes in body position. Orthostatic pressure changes from changes in body position have been shown to significantly influence mean pressures in sheep, while minimal patterns in pulse pressure suggest potential independence between body position and pulse, at least at moderate tilts in posture. These relationships suggest that pulse pressure may be considered as constant and could be exploited as control parameter. Transfer function analyses quantify previously unquantified physiological insights: there exists a general compliant, lowpass theme to the arterio-craniospinal, arterio-venous, and arterio-abdominal systems. However, there are also overlain higher-order dynamics present.

## Data Availability

All relevant in-vivo data used in this study can be found open access using the following link: 10.3929/ethz-b-000623698.
